# Increasing Trends of Clostridium difficile Infection in Hospitalized Young Patients: A Study of the National Inpatient Sample From 2007 to 2017

**DOI:** 10.7759/cureus.29497

**Published:** 2022-09-23

**Authors:** Tsu Jung Yang, Achint A Patel, Jassimran Singh, Vinay Jahagirdar, Dhanshree Solanki, Bharati Nikhare, Nishi Harwani, Ruchir Goswami, Hiteshkumar Devani, Prakash Maiyani, Dharmeshkumar V Moradiya, Maheshkumar Desai, Salman Muddassir

**Affiliations:** 1 Medicine, MultiCare Good Samaritan Hospital, Puyallup, USA; 2 Internal Medicine, HCA Healthcare/University of South Florida Morsani College of Medicine Graduate Medical Education/Oak Hill Hospital, Brooksville, USA; 3 Medicine, Institute of Medical Sciences, Banaras Hindu University, Varanasi, IND; 4 Internal Medicine, University of Missouri–Kansas City School of Medicine, Kansas City, USA; 5 Hospital Administration, Rutgers University, New Brunswick, USA; 6 Medicine, St. John’s Episcopal Hospital, Far Rockaway, USA; 7 Medicine, State University of New York Downstate Medical Center, Brooklyn, USA; 8 Epidemiology and Public Health, Icahn School of Medicine at Mount Sinai, New York, USA; 9 Dental Medicine, University of Pittsburgh School of Dental Medicine, Pittsburgh, USA; 10 Internal Medicine, Gold Coast University Hospital, Southport, AUS; 11 Internal Medicine, St. John of God Murdoch Hospital, Murdoch, AUS; 12 Internal Medicine, Hamilton Medical Center, Medical College of Georgia/Augusta University, Dalton, USA

**Keywords:** cdi hospitalizations, outcomes, trend, young population, clostridium difficile

## Abstract

Background

*Clostridium difficile* infection (CDI) is one of the rising public health threats in the United States. It has imposed significant morbidity and mortality in the elderly population. However, the burden of the disease in the young population is unclear. This study aimed to identify hospitalization trends and outcomes of CDI in the young population.

Methodology

We obtained data from the National (Nationwide) Inpatient Sample (NIS) for hospitalizations with CDI between 2007 and 2017. We used the International Classification of Diseases Ninth Edition-Clinical Modification (ICD-9-CM) and ICD-10-CM to identify CDI and other diagnoses of interest. The primary outcome of our study was to identify the temporal trends and demographic characteristics of patients aged less than 50 years old hospitalized with CDI. The secondary outcomes were in-hospital mortality, length of hospital stay (LOS), and discharge dispositions. We utilized the Cochran Armitage trend test and multivariable survey logistic regression models to analyze the trends and outcomes.

Results

From 2007 to 2017, CDI was present among 1,158,047 hospitalized patients. The majority (84.04%) of the patients were ≥50 years old versus 15.95% of patients <50 years old. From 2007 to 2017, there was a significant increase in CDI among <50-year-old hospitalized patients (12.6% from 2007 to 18.1% in 2017; p < 0.001). In trend analysis by ethnicities, among patients <50 years old, there was an increasing trend in Caucasians (63.9% versus 67.9%; p < 0.001) and Asian females (58.4% versus 62.6%; p < 0.001). We observed an increased trend of discharge to home (91.3% vs 95.8%; p < 0.001) in association with a decrease in discharge to facility (8.3% vs 4%; p < 0.001). The average LOS from 2007 to 2017 was 5 ± 0.03 days, which remained stable during the study period.

Conclusions

The proportion of young (<50 years old) hospitalized patients with CDI has been steadily increasing over the past decade. Our findings might represent new epidemiological trends related to non-traditional risk factors. Future CDI surveillance should extend to the young population to confirm our findings, and the study of emerging risk factors is required to better understand the increasing CDI hospitalization in the young population.

## Introduction

*Clostridium difficile* infection (CDI) is the inflammation and damage of colonic mucosa caused by toxins produced from *C. difficile* colonization. The infection often manifests as watery diarrhea (≥three loose stools in 24 hours), abdominal pain, and cramping, which can be complicated by fulminant colitis resulting in toxic megacolon, intestinal perforation, septic shock, and multiorgan failure. The infection is transmitted by the fecal-oral route when the normal colonic mucosa is destroyed by antibiotics, especially in association with healthcare [1]. CDI represents the most common cause of healthcare-acquired infections in the United States [2]. Hall et al. reported that CDI was the leading cause of mortality associated with gastroenteritis in the United States, with the mortality rate increasing fivefold from 1999 to 2007 [3]. In 2013, a Centers for Disease Control and Prevention (CDC) report regarding “Antibiotic Resistance Threats in the United States” categorized *C. difficile* as an urgent threat and implemented several measures, including antibiotic stewardship, stringent infection control to contain the spread, pay-for-performance programs, and frequent healthcare facility disinfection practices. Despite multimodal approaches, it contributed to 223,900 estimated cases of hospitalized patients, 12,800 estimated deaths, and 1 billion US dollars of estimated attributable healthcare costs in 2017 [4]. Although it is well known that elderly adults (>65 years of age) have a higher rate of CDI, higher mortality, and longer length of hospital stay (LOS) compared to the younger population [5,6], the most recent age-specific incidence and trends of CDI, especially in the younger population, remain unclear. The goal of our study is to evaluate the current epidemiology and burden of CDI in young, hospitalized patients between 18 and 50 years of age. From 2001 to 2010, there was a twofold increase in the rates of CDI hospitalizations [7]. The rise was linked to the emergence of a new virulent strain, North American pulsed-field gel electrophoresis type 1 (NAP1)/ribotype 027 [6,8]. To control the outbreak, the CDC declared a nationwide urgent threat to CDI and initiated widespread preventive measures such as antibiotic stewardship and stringent infection controls. Among various risk factors, age is one of the most important factors for acquiring CDI. Aging is associated with frequent exposure to the healthcare system, long-term care facility residence, and age-related changes in physiologic function such as diminished immune responses and alterations in the gut microbiome [5,9]. Although the elderly population experiences higher mortality and longer median length of hospital stay when compared to young adults, the relationship between age and CDI may not be as linear as it is generally believed. Olsen et al. suggested that acute infections, particularly septicemia and pneumonia, healthcare exposures such as emergency hospitalization and skilled nursing facility stay, frailty indicators, and acute noninfectious conditions such as acute myocardial infarction and gastrointestinal bleeding were more important predictors of CDI rather than age alone in the elderly population [10]. However, the study was limited to Medicare beneficiaries and the elderly population. Therefore, this result is not generalizable to young adults, particularly those with poorer overall health status.

## Materials and methods

Data source

We extracted our study cohort from the National (Nationwide) Inpatient Sample (NIS) of the Healthcare Cost and Utilization Project (HCUP), Agency for Healthcare Research and Quality (AHRQ) [11]. The NIS is one of the largest all-payer publicly available databases on inpatient discharges from US hospitals maintained by the AHRQ. The NIS approximates a 20% stratified sample of discharges from US community hospitals, excluding rehabilitation and long-term acute care hospitals, and contains more than seven million hospitalizations annually. With the established weights provided in the NIS, this data can represent the standardized US population to obtain national estimates with high accuracy.

Study population and design

We queried the 2007-2017 NIS database using the International Classification of Diseases Ninth Edition/Tenth Edition-Clinical Modification (ICD-9/10-CM) diagnosis codes for CDI as primary and secondary diagnosis fields. These codes have been used by previously published articles from administrative databases such as iNIS [6,11]. We extracted demographics, hospital-level characteristics (geographical region, size, and teaching status), and patient-level characteristics, supplied as part of the NIS [12,13]. We estimated comorbidities using Elixhauser comorbidity software and mortality risk using the validated All Patient Refined Diagnosis Related Groups (APR-DRGs) mortality score, which are also supplied by HCUP tools and software [13,14]. We identified specific concurrent medical conditions and procedures of interest using ICD-9/10-CM diagnosis and procedure codes. We classified patients <50 years of age as “young” and ≥50 years of age as “old.”

Statistical analysis

To establish the trend, we calculated the proportion of hospitalized patients with CDI in the age group <50 versus ≥50 years. We then calculated the proportional trends of CDI by different demographics to estimate temporal changes during the study period in the age group <50 years. To study the CDI outcomes, we divided hospitalization disposition into three categories, namely, discharge to home, discharge to facilities, and in-hospital mortality. We used the Cochran Armitage trend test and survey logistic regression for trend analysis. We utilized SAS 9.3 (SAS Institute, Cary, NC, USA) for all analyses, and included designated weight values to produce nationally representative estimates [12,15]. For regression models, we used survey procedures to account for the inherent survey design of the NIS to produce more robust estimates [16]. We considered a two-tailed p-value of <0.05 as statistically significant.

## Results

Temporal trends of *Clostridium difficile* infection in hospitalized young patients

**Figure 1:**
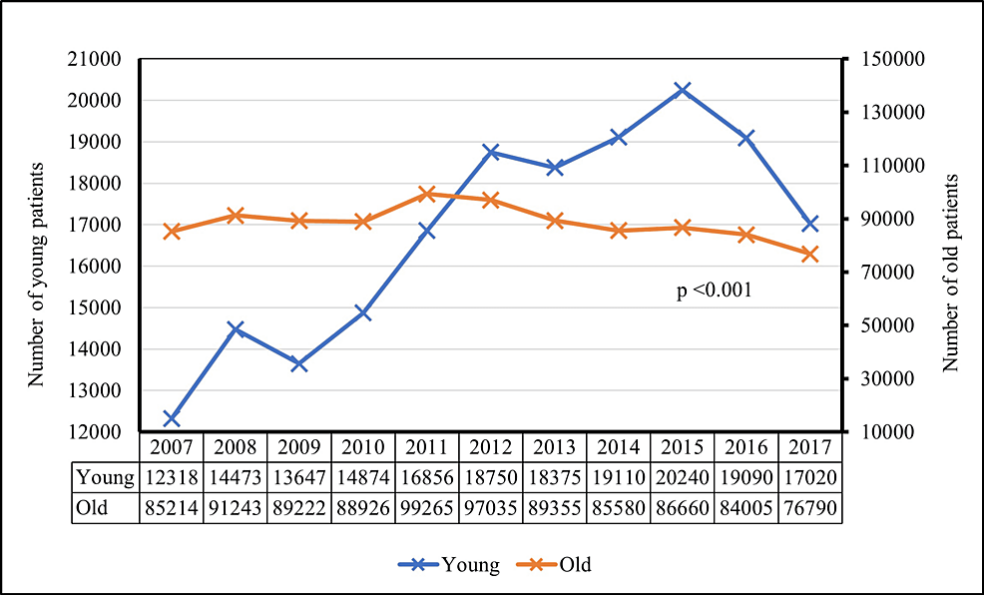
Temporal trends of CDI in young versus old patients. CDI = *Clostridium difficile* infection

**Figure 2:**
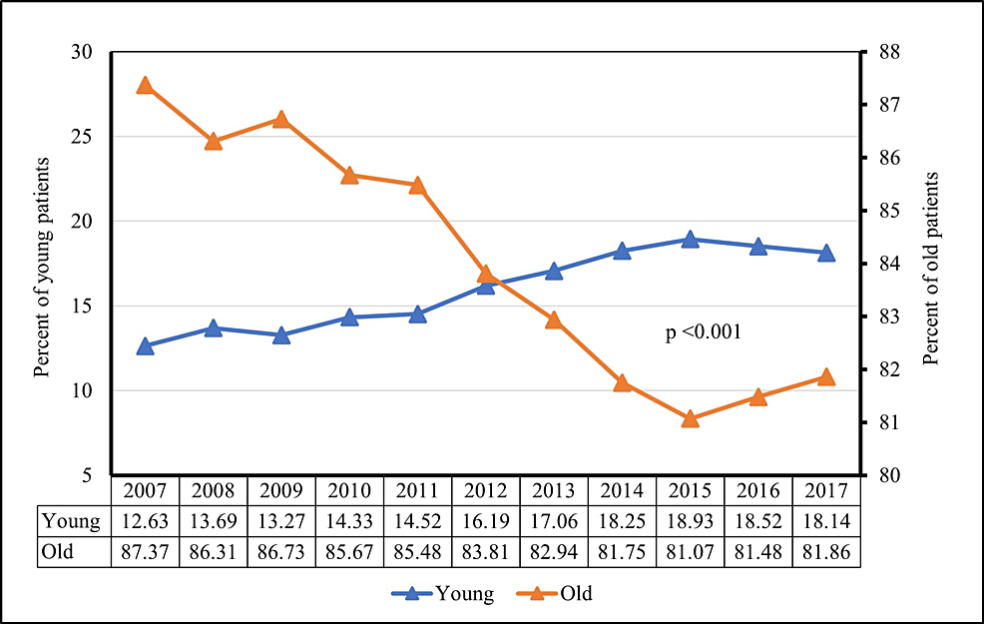
Proportional trends of CDI in young versus old patients. CDI = *Clostridium difficile* infection

The number of total hospitalized patients with CDI were 1,158,047 from 2007 to 2017. Although the number of hospitalizations was high among patients aged ≥50 years compared to those aged <50 years (84.0% vs. 15.6%, p < 0.001), there was a considerable increase in the proportion of hospitalized patients <50 years of age with CDI from 2007 to 2017 (12.6% vs. 18.1%, p < 0.001). In contrast, there was a decline in the proportion of hospitalized patients ≥50 years of age with CDI from 2007 to 2017 (87.4% vs. 81.9%, p < 0.001). Figure 1 and Figure 2 illustrate the trend of hospitalized patients with CDI from 2007 to 2017.

Temporal trends of Clostridium difficile infection in hospitalized young patients by demographics

Our data showed that the proportion of hospitalized adults aged 18-35 years with CDI increased from 31.8% in 2007 to 37.0% in 2017 (p < 0.001). The gender-wise proportional trends remained stable over the years, with females accounting for around 64.0% of the total patients with CDI between 2007 and 2017 (p = 0.378). The proportion of hospitalized Caucasian patients with CDI increased from 63.9% in 2007 to 67.9% in 2017. However, among African Americans, the trend declined from 18.5% in 2007 to 15.9% in 2017. Of note, the proportional trend of hospitalized Asian females with CDI increased from 58.4% in 2007 to 62.6% in 2017 (p < 0.001). The detailed proportional trends are depicted in Table 1.

**Table 1:** Temporal trends of CDI in younger patients by demographics. CDI = *Clostridium difficile* infection

Characteristics	2007	2008	2009	2010	2011	2012	2013	2014	2015	2016	2017	P-value
Total hospitalizations	12,318	14,473	13,647	14,874	16,856	18,750	18,375	19,110	20,240	19,090	17,020	
Age groups												<0.001
18–35 years	31.8	33.1	32.7	35.5	37.1	37.6	38.5	38.1	38.8	39.2	37.1	
35–49	68.2	66.9	67.3	64.5	62.9	62.4	61.6	61.9	61.2	60.8	62.9	
Gender												0.378
Male	36.0	37.1	35.9	35.9	34.6	36.4	35.7	34.9	35.5	34.2	36.3	
Female	64.1	62.9	64.1	64.1	65.4	63.6	64.3	65.1	64.5	65.8	63.8	
Ethnicity												<0.001
Caucasian	63.9	68.8	68.8	68.7	68.2	68.8	69.3	69.0	66.6	66.9	67.9	
African American	18.5	15.8	14.7	18.1	16.6	16.5	16.1	16.1	18.3	16.7	15.9	
Hispanic	11.7	10.8	11.5	9.1	10.9	9.6	10.1	10.5	10.8	11.5	10.9	
Asians/Others	6.0	4.6	5.0	4.2	4.4	5.1	4.5	4.4	4.4	4.9	5.3	
Females by ethnicity												<0.001
Caucasian	65.0	64.0	65.9	65.8	66.2	66.0	65.5	66.1	67.3	67.3	65.4	
African American	66.0	63.5	59.3	60.4	63.2	60.3	60.0	65.8	60.5	64.3	60.6	
Hispanic	59.9	58.6	57.5	60.7	66.1	55.9	63.3	62.4	55.0	64.2	59.2	
Asians/Others	58.4	62.8	61.5	62.2	61.5	56.1	58.6	57.8	63.7	59.6	62.6	

Outcomes of Clostridium difficile infection in hospitalized young patients

**Figure 3:**
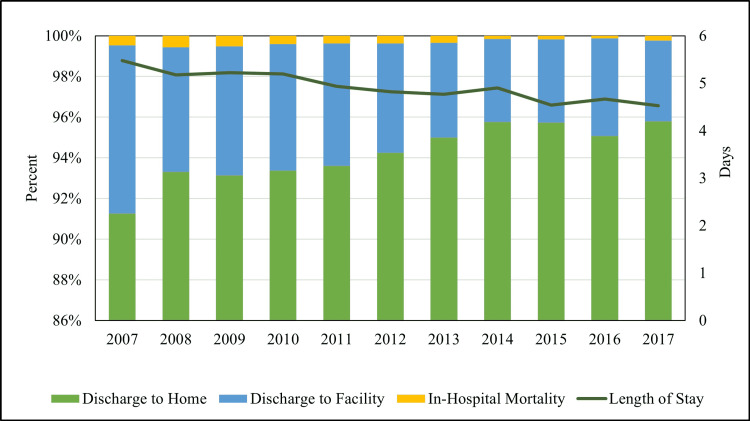
Temporal trends of discharge disposition in young patients with CDI. CDI = *Clostridium difficile* infection

The overall number of patients aged <50 years hospitalized with CDI between the years 2007 and 2017 was 184,634. The number of patients discharged home consistently increased from 91.3% in 2007 to 95.8% in 2017 (p < 0.001) (Figure 3). The number of patients discharged to the facility persistently decreased from 8.3% in 2007 to 4% in 2017 (p < 0.001) (Figure 2). The in-hospital mortality in patients with CDI decreased from 0.5% in 2007 to 0.1% in 2016 (p < 0.001). The average LOS in the hospital for patients with CDI was 5 ± 0.03 days which remained stable during the study period.

## Discussion

We found that the proportion of young, hospitalized patients with CDI notably increased from 12.6% to 18.1% during 2007-2017 (p < 0.001). Although the studied age group was not identical, Pechal et al. similarly noted that CDI incidence increased from 2001 to 2010 in adult discharges between 18 and 74 years old [5]. One probable reason for our finding is the increasing incidence of community-acquired *C. difficile* infection (CA-CDI). CA-CDI is defined by symptom onset within the community or ≤48 hours after hospital admission with symptom onset more than 12 weeks since the last healthcare facility exposure [17,18]. Ofori et al. reported that in one-third of CA-CDI cases, there was no association with a prior or recent history of antibiotic prescription, and CA-CDI represented approximately half of all CDI cases in the United States [19]. According to a regional study by Lewis et al., the adjusted annual incidence rate of CA-CDI increased twofold from 2011 to 2015 [20]. It tends to affect younger female patients with lesser exposure to antibiotics compared to hospital-acquired CDI [21]. Especially in patients between 18 and 35 years of age, CDI increased from 31.81% in 2007 to 37% in 2017 (p < 0.001). On the other hand, we saw a decrease in incidence among patients aged 35-49 years from 68.19% in 2007 to 62.9% in 2017 (p < 0.001). Other hypotheses to our findings include a shift in epidemiology, the emergence of new hypervirulent strains, an increase in asymptomatic carriers, and zoonotic transmission. A study by Turner et al. found that CA-CDI cases increased over time from 2013 to 2017 (odds ratio (OR) = 1.010 per month; 95% confidence interval (CI) 1.006-1.015; p < 0.001) [22]. In contrast, the most recent report from the CDC Emerging Infections Program (EIP) estimated that the prevalence of CA-CDI over the last decade has not changed although hospital cases have been decreasing [8]. The extrapolation of hospital data to community infections can be confounding. The actual incidence could have been underestimated because not all infected patients in the communities presented to the hospital. CDI is not a reportable illness and the nucleic acid amplification test (NAAT) cannot differentiate between active infection and colonization, which further makes community surveillance challenging. Depending on case definitions, a substantial proportion of CA-CDI cases have previous healthcare contacts and can be classified as HA-CDI. In addition, because most published studies were conducted in hospital settings, currently available data cannot represent the actual prevalence of CA-CDI.

As evident by our study, the number of hospitalized patients aged ≥50 years with CDI decreased from 87.4% in 2007 to 81.9% in 2017 (p < 0.001). Our result is in accordance with the projected burden of CDI from a study funded by the CDC. The study concluded that the adjusted estimate of the total burden of CDI decreased by 24% (95% CI = 6 to 36) from 2011 to 2017 [8]. A retrospective, multicenter cohort study of 43 regional community hospitals that participated in the Duke Infection Control Outreach Network (DICON) from 2013 to 2017 also showed a modest decrease in HA-CDI after accounting for the increased use of NAAT [22]. NAAT is more sensitive than traditional enzyme immunoassay testing and can result in the overestimation of CDI. Regardless of the increased CDI in the young population, our study showed that the elderly populations were still accountable for 84% of total hospitalized patients with CDI in the past decade.

The gender trends of CDI in the young population remained stable during our study period (p = 0.378). CDI was more common in females, around 64%, but it did not reach statistical significance. Similar to the elderly population, CDI was more common in Caucasians; however, we noted an increasing proportional trend in Asian females from 58.4% in 2007 to 62.6% in 2017 (p < 0.01). Especially, Asian populations have seen faster growth than any other race group in the United States [23,24]. According to a retrospective study of the Medicare Patient Safety Monitoring System database from 2009 to 2011, Hispanic and Asian patients had higher rates of hospital-acquired infections than Caucasian patients after adjusting for age, gender, and comorbidities, especially hospital-acquired CDI [25]. Yang et al. reported that Caucasian patients had an increased risk of CDI, but the majority of analyzed studies were heterogenous and had significant methodological limitations [26]. In contrast, racial differences in CDI might not exist if racial disparities in healthcare are eliminated. This was evident in a study on skilled nursing facility residents by Mao et al. who noted that the apparent racial differences in CDI risks may represent healthcare access disparities, rather than genotypic differences [27]. Nonetheless, our findings suggest that the demographics of CDI are changing.

Our study also noted that the average hospital LOS for CDI in the young population was 5 ± 0.03 days and remained stable over the study period. Comparatively, the median hospital LOS for adults (18-64 years old) from 2001 to 2010 was seven days (4-14 days; p < 0.0001) according to a similar study by Pechal et al. [5]. Although the reason is not clear, we saw a statistically significant decrease in CDI discharges to facilities and in-hospital mortality from 2007 to 2017. Patients discharged to facilities are usually sicker with multiple comorbidities compared to home discharges, so it is reassuring to see the downward trend. The young population usually has fewer comorbid conditions than the elderly population, which may explain lower LOS and in-hospital mortality. The new advent of CDI treatment such as fecal microbiota transplantation could have played a role as well [28].

Our study has several limitations. First, we acquired data using ICD-9/10-CM codes, which may not be ideal for disease surveillance. Although the administrative codes may overestimate CDI compared to toxin assay results [29], it is a valuable tool to measure the overall burden of CDI because it is readily available and represents a nationwide population. Second, we could not differentiate between active disease and colonization while interpreting our data, especially with the increasing use of NAAT in the United States over the past decade. Third, the probable causes and explanations of our findings were not studied owing to the limitations of our database. Although we hypothesized that our finding may be related to increasing CA-CDI cases, our data were hospital-based and could not represent community infections. Future studies are required to elucidate the relationship between CA-CDI and CDI hospitalizations in the young population. Despite the limitations, our database is representative of the US population. We were able to study the trends of CDI in racial and ethnic minorities because of the large sample size. Our study is also the first to highlight the trends of CDI in the young population. In addition, the results of our study emphasize the need to report age-specific incidence rates and the importance of including racial and ethnic minorities in epidemiological studies.

## Conclusions

Our study emphasizes that the proportion of young (<50 years of age) hospitalized patients with CDI increased over the past decade. While the main risk factors for CDI include old age and antibiotic use, non-traditional risk factors could have contributed to our findings. We highlight the importance of including the young population in future CDI surveillance and the study of emerging risk factors.

## References

[1] Czepiel J, Dróżdż M, Pituch H (2019). Clostridium difficile infection: review. Eur J Clin Microbiol Infect Dis.

[2] Magill SS, Edwards JR, Bamberg W (2014). Multistate point-prevalence survey of health care-associated infections. N Engl J Med.

[3] Hall AJ, Curns AT, McDonald LC, Parashar UD, Lopman BA (2012). The roles of Clostridium difficile and norovirus among gastroenteritis-associated deaths in the United States, 1999-2007. Clin Infect Dis.

[4] (2020). Prevention CfDCa: antibiotic resistance threats in the United States, 2019. http://www.cdc.gov/drugresistance/pdf/threats-report/2019-ar-threats-report-508.pdf.

[5] Pechal A, Lin K, Allen S, Reveles K (2016). National age group trends in Clostridium difficile infection incidence and health outcomes in United States Community Hospitals. BMC Infect Dis.

[6] Solanki D, Kichloo A, El-Amir Z, Dahiya DS, Singh J, Wani F, Solanki S (2021). Clostridium difficile infection hospitalizations in the United States: insights from the 2017 National Inpatient Sample. Gastroenterology Res.

[7] Reveles KR, Lee GC, Boyd NK, Frei CR (2014). The rise in Clostridium difficile infection incidence among hospitalized adults in the United States: 2001-2010. Am J Infect Control.

[8] Guh AY, Mu Y, Winston LG (2020). Trends in U.S. burden of Clostridioides difficile infection and outcomes. N Engl J Med.

[9] Asempa TE, Nicolau DP (2017). Clostridium difficile infection in the elderly: an update on management. Clin Interv Aging.

[10] Olsen MA, Stwalley D, Demont C, Dubberke ER (2018). Increasing age has limited impact on risk of Clostridium difficile infection in an elderly population. Open Forum Infect Dis.

[11] Petersen MR, Cosgrove SE, Klein EY (2021). Clostridioides difficile prevalence in the United States: National Inpatient Sample, 2016 to 2018. Open Forum Infect Dis.

[12] (2020). Overview of HCUP. https://www.ahrq.gov/data/hcup/index.html.

[13] (2020). NIS description of data elements. https://www.hcup-us.ahrq.gov/db/nation/nis/nisdde.jsp.

[14] (2021). Elixhauser Comorbidity Software, Version 3.7. https://www.hcup-us.ahrq.gov/toolssoftware/comorbidity/comorbidity.jsp.

[15] (2020). Trend weights for HCUP NIS data. https://www.hcup-us.ahrq.gov/db/nation/nis/trendwghts.jsp.

[16] (2021). HCUP methods series. https://www.hcup-us.ahrq.gov/reports/methods/methods.jsp.

[17] Gupta A, Khanna S (2014). Community-acquired Clostridium difficile infection: an increasing public health threat. Infect Drug Resist.

[18] Kim G, Zhu NA (2017). Community-acquired Clostridium difficile infection. Can Fam Physician.

[19] Ofori E, Ramai D, Dhawan M, Mustafa F, Gasperino J, Reddy M (2018). Community-acquired Clostridium difficile: epidemiology, ribotype, risk factors, hospital and intensive care unit outcomes, and current and emerging therapies. J Hosp Infect.

[20] Lewis SS, Baker AW, Moehring RW, Sexton D, Anderson DJ (2016). Increasing incidence of community-acquired Clostridium difficile infections among hospitalized patients. Open Forum Infect Dis.

[21] Khanna S, Pardi DS, Aronson SL (2012). The epidemiology of community-acquired Clostridium difficile infection: a population-based study. Am J Gastroenterol.

[22] Turner NA, Grambow SC, Woods CW, Fowler VG Jr, Moehring RW, Anderson DJ, Lewis SS (2019). Epidemiologic trends in Clostridioides difficile infections in a regional community hospital network. JAMA Netw Open.

[23] (2021). The Asian Population. https://www.census.gov/library/publications/2012/dec/c2010br-11.html.

[24] Mui P, Bowie JV, Juon HS, Thorpe RJ Jr (2017). Ethnic group differences in health outcomes among Asian American men in California. Am J Mens Health.

[25] Bakullari A, Metersky ML, Wang Y (2014). Racial and ethnic disparities in healthcare-associated infections in the United States, 2009-2011. Infect Control Hosp Epidemiol.

[26] Yang S, Rider BB, Baehr A, Ducoffe AR, Hu DJ (2016). Racial and ethnic disparities in health care-associated Clostridium difficile infections in the United States: state of the science. Am J Infect Control.

[27] Mao EJ, Kelly CR, Machan JT (2015). Racial differences in Clostridium difficile infection rates are attributable to disparities in health care access. Antimicrob Agents Chemother.

[28] Cheng YW, Phelps E, Nemes S (2020). Fecal microbiota transplant decreases mortality in patients with refractory severe or fulminant Clostridioides difficile infection. Clin Gastroenterol Hepatol.

[29] Dubberke ER, Butler AM, Yokoe DS (2010). Multicenter study of surveillance for hospital-onset Clostridium difficile infection by the use of ICD-9-CM diagnosis codes. Infect Control Hosp Epidemiol.

